# Substance P/ Neurokinin-1 Receptor, Trigeminal Ganglion, Latency, and Coronavirus Infection-Is There Any Link?

**DOI:** 10.3389/fmed.2021.727593

**Published:** 2021-11-18

**Authors:** Riffat Mehboob, Maher Kurdi, Ahmed Bamaga, Njoud Aldardeir, Hisham Nasief, Leena H. Moshref, Taghreed Alsinani, Almotasimbellah O. Rayes, Reem H. Jabbad

**Affiliations:** ^1^Faculty of Allied Health Sciences, The University of Lahore, Lahore, Pakistan; ^2^Lahore Medical Research Center, LLP, Lahore, Pakistan; ^3^Department of Pathology, Faculty of Medicine, King Abdulaziz University, Rabigh, Saudi Arabia; ^4^Neurology Division, Department of Pediatrics, Faculty of Medicine, King Abdulaziz University, Jeddah, Saudi Arabia; ^5^Faculty of Education, University of Ottawa, Ottawa, ON, Canada; ^6^Department of Obstetrics and Gynecology, Faculty of Medicine, Jeddah, Saudi Arabia; ^7^Department of Surgery, Doctor Soliman Fakeeh Hospital, Jeddah, Saudi Arabia; ^8^Division of Neurosurgery, King Fahad General Hospital, Jeddah, Saudi Arabia; ^9^Department of Obstetrics and Gynecology, Faculty of Medicine, King Abdulaziz University, Jeddah, Saudi Arabia; ^10^Department of Medicine, King Fahad Armed Forces Hospital, Jeddah, Saudi Arabia

**Keywords:** coronavirus, Substance P/ Neurokinin-1 Receptor, respiratory illness, infectious disease, trigeminal ganglion

## Abstract

Novel Severe Acute Respiratory Syndrome-Corona Virus-2 infection (SARS-CoV-2) is an acute respiratory and infectious disease. This perspective aims to provide a basic understanding of the inflammation caused by SARS-CoV-2 and its relation to the trigeminal ganglion (TG). The virus enters through the mucous membranes of the orofacial region and reaches the TG, where it resides and takes control of its peptides including Substance P (SP). SP is the main neuropeptide, neuromodulator, and neuro-hormone of TG, associated with nociception and inflammation under noxious stimulus. SP release is triggered and, consequently, affects the immune cells and blood vessels to release the mediators for inflammation. Hence, cytokine storm is initiated and causes respiratory distress, bronchoconstriction, and death in complicated cases. Neurokinin-1 Receptor (NK-1R) is the receptor for SP and its antagonists, along with glucocorticoids, may be used to alleviate the symptoms and treat this infection by blocking this nociceptive pathway. SP seems to be the main culprit involved in the triggering of inflammatory pathways in SARS-CoV-2 infection. It may have a direct association with cardio-respiratory rhythm, sleep-wake cycle, nociception, and ventilatory responses and regulates many important physiological and pathological functions. Its over-secretion should be blocked by NK-1R antagonist. However, experimental work leading to clinical trials are mandatory for further confirmation. Here, it is further proposed that there is a possibility of latency in SARS-CoV-2 virus infection if it is acting through TG, which is the main site for other viruses that become latent.

## Introduction

**Figure 1:**
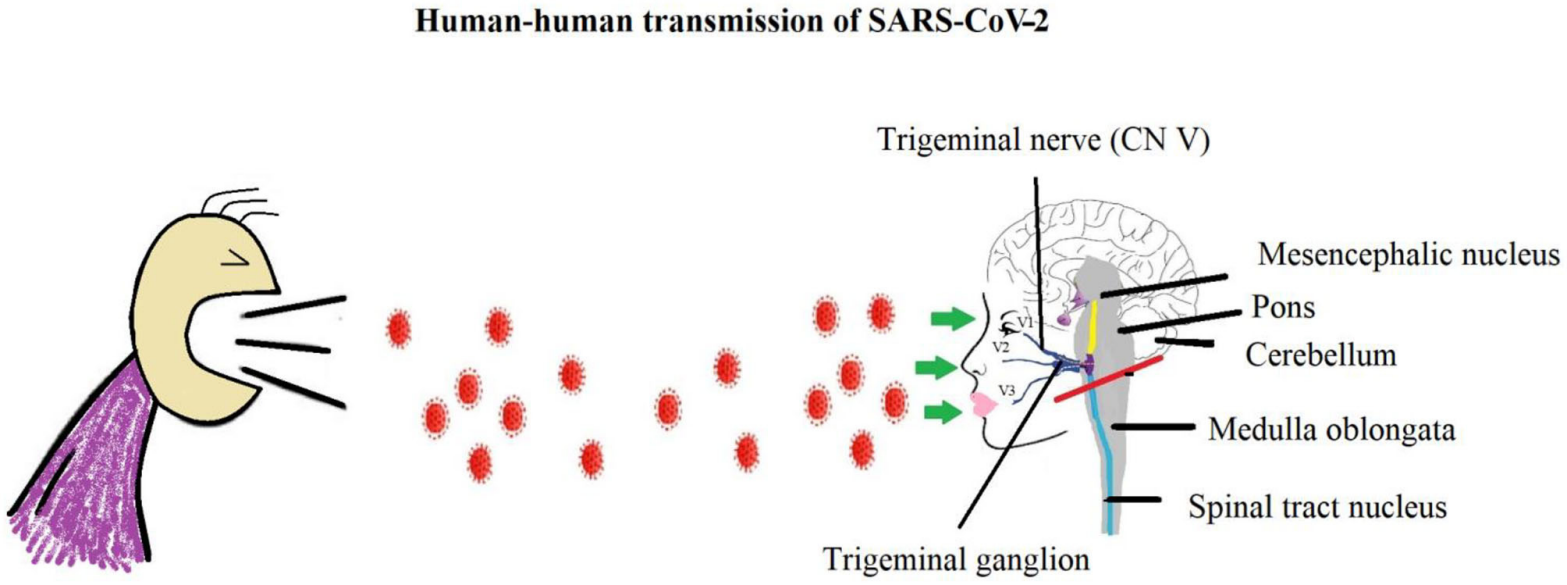
SARS-CoV-2 transmission from one human to another and Trigeminal innervation in ophthalmic, maxillary, and mandibular region.

Severe Acute Respiratory Syndrome-Corona Virus-2 (SARS-CoV-2) is responsible for causing Corona virus infection (COVID-19) which was first reported in Wuhan, China in December 2019 but went on to affect the whole world (1). It has become a pandemic, as declared by WHO in March, 2020 (2), and shattered the global economy in a very short span of time. The common symptoms include fever, cough, fatigue, acute respiratory distress syndrome, and body aches (3, 4). Complicated cases may undergo respiratory failure or even death (5). Common and initial symptoms of COVID-19 infection include sore throat, loss of sense of smell and taste, pain in eyes, headache, and flu (3). Similar functions are carried out by SP once it is released from the trigeminal ganglion *via* trigeminal nerve (TrN) when triggered by a nociceptive stimulus. It provides somatosensory innervation to the orofacial region. So any alteration in its secretion in response to viral infection may result in symptoms in the orofacial region (6) (Figure 1).

SP may be responsible for the initiation of inflammatory pathways and should be explored further. It aggravates the condition due to its over secretion by TG neurons which affects the immune cells as well as other cells in the respiratory tract to release the mediators for cytokine storming which may be responsible for further complications. The ventilatory role of SP is well-established (7). There may be a less unlikely possibility of latency in SARS-CoV-2 and this virus may reach the TG *via* TrN in the eyes, nose, and mouth and controls the release of peptides including SP. The virus may remain in a latent form in TG and may reactivate at any time, either causing infection leaving the patient asymptomatic in this condition. It may remain dormant or latent within a cell and the replication of virus ceases after initial infection (8). Viral genome may stay in the cell and may get reactivated at any time (9). Reactivation may occur due to stress or UV (10). The viruses may be retained in the cells of the host after initial infection despite antibodies against it in the blood (11).

In my recently published study, I have proposed that SP/ Neurokinin 1-Receptor may serve as a potential therapeutic strategy against COVID-19 infection (12). I have also conducted a clinical trial and administered Neurokinin 1-Receptor antagonist, Aprepitant, in the interventional group and the results were encouraging. The interventional group showed more recovery, decreased C-reactive protein (which is an inflammatory marker), and improved platelet levels (13). Here, in this current study, I suggest a potential threat of latency in SARS-CoV-2 and we should investigate this phenomenon as well.

## Substance P and Neurokinin−1 Receptor

SP is a “brain-gut” hormone and the first inflammatory neuropeptide discovered in 1931 by Euler and Gaddum (14). It belongs to the Tachykinin (TK) family of proteins, which is the largest of protein families with ~40 members. It has 11 amino-acids and is a neuromodulator, the most potent vasodilator, and a neurotransmitter, involved in signal transmission, encoded by Tachykinin-1 *(TAC-1)* gene (15). SP is released in the TG by the fifth cranial nerve (CN V) endings, known as trigeminal nerve (TrN). TrN provides the main afferent pathways for the transmission of nociception and pain, controls the physiological mechanisms in the orofacial region, including eyes, nose, mouth, and is associated with their physiological and pathological functions. SP is localized in the respiratory nuclei of the respiratory network which controls the ventilatory functions, cardiac functions, and sleep-wake cycle (6, 7, 16, 17). They control the function of eyes, mouth, nose, tongue, lips, facial muscle, mastication, gustation, and olfaction under normal circumstances. SP is present in perivascular neural plexuses of lung, skin, and brain. So, its effects are not only limited to the nervous system but are wide in distribution and expression (17). Respiratory rhythm regulation is the main role of SP, which has been evidenced through many studies (16, 18). SP along with serotonin is found to innervate the medullary motoneurons involved in upper airways (7). It was also found to be a sensory neurotransmitter in the laryngeal afferent system (19).

Neurokinin-1 Receptor (NK-1R) is the receptor for SP and most of the functions of SP are elicited only after binding with its receptor. Both SP and NK-1R are abundant in the central nervous system (CNS), peripheral nervous system (PNS), and enteric system (20). It also regulates the immune system and cardio-vasculatory system (21, 22). SP is a neurotransmitter but can also affect distant cells by acting as a modulator or hormone functioning in an autocrine, endocrine, and paracrine manner. It is released from non-neuronal cells as well such as immune cells (23). It is associated with respiratory inflammation e.g., asthma and chronic obstructive pulmonary disease (COPD) (24). NK-1R is a 7-transmembrane, G-protein coupled receptor with 407 amino-acids. It is located on several cells in the circulatory, digestive, respiratory, and immune systems (15). It is mainly present in the brainstem region where it controls key functions such as respiration and cardiac control after binding with SP (25).

## Trigeminal Nociceptive Pathways

**Figure 2:**
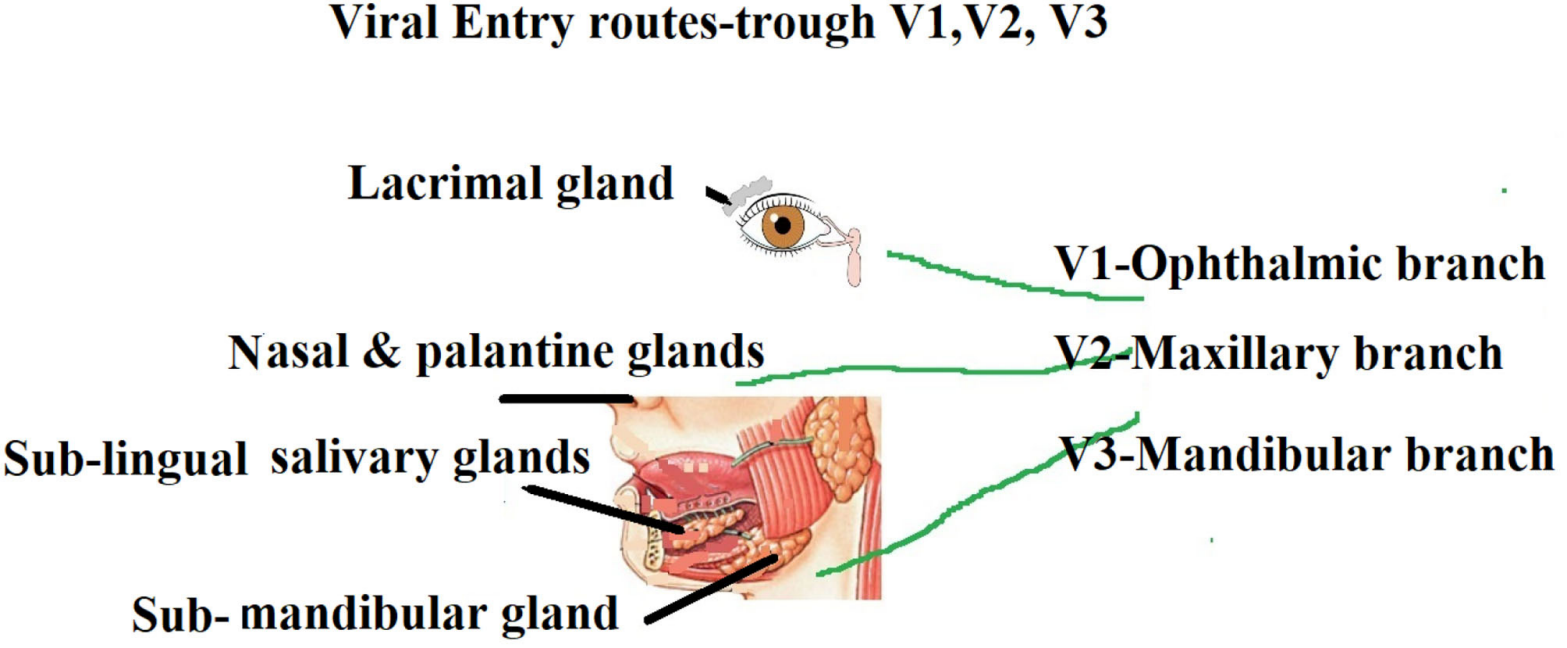
SARS-CoV-2 entry through orofacial structures.

Any noxious stimulus from the orofacial structures, such as the eye, nose, or mouth, is mainly transmitted by the TrN (Figure 1). TG neurons produce SP and CGRP which are largely involved in neuromodulation as well as in inflammation and nociception (26). The primary afferent neurons of the TrN are mainly located in the TG and partially in the mesencephalic trigeminal nucleus in the brainstem. The TrN consists of three branches: the ophthalmic (V1), maxillary (V2), and mandibular (V3) nerves. Each provides innervation to their respective regions of the head (27) (Figures 1, 2).

**Figure 3:**
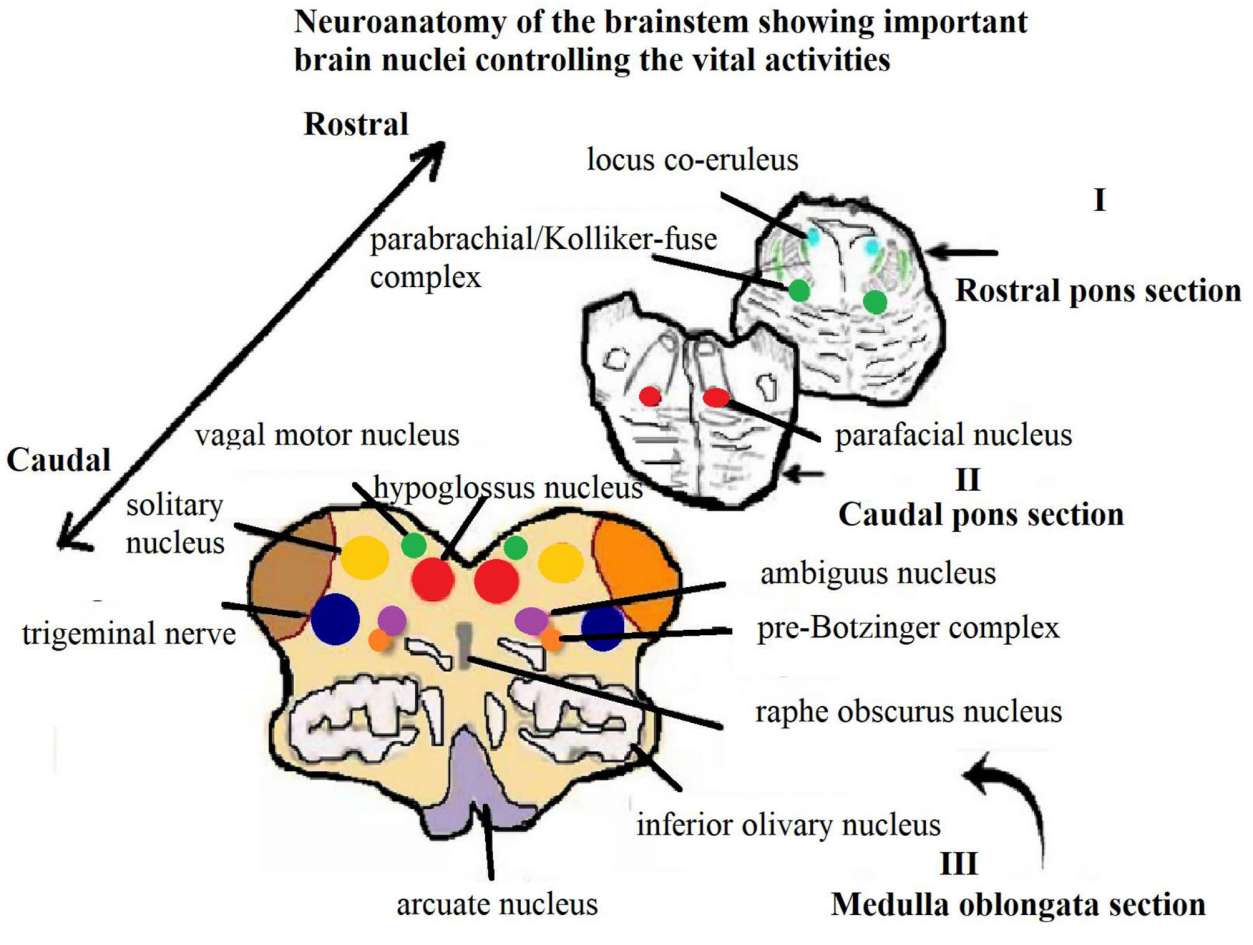
Schematic section of the main histological sections from the brainstem showing Trigeminal nerve and solitary nucleus, which are the main source of Substance P secretion and stimulation (28).

Pain or any other noxious stimulus, such as SARS-CoV-2, activates the nociceptors which are the free nerve endings of trigeminal sensory afferents. These sensory nerve fibers are myelinated or non-myelinated C-fibers and their cell bodies reside in the TG (6, 27). These signals are carried *via* afferent fibers to the trigeminal spinal caudalis (Vc) nucleus of the brainstem. Here, they synapse with the second order neurons that project to the somatosensory and limbic cortices *via* the thalamus. Inflammation of orofacial tissues that are innervated by the TrN can modify the activity of trigeminal afferent neurons, consequently causing ectopic firing and raised sensitivity of noxious stimuli. Sensitization is facilitated by many mediators such as neurotrophic factors or neuropeptides at nerve endings, such as SP, CGRP, and serotonin (26). SP and CGRP levels increase in TG and TrN (Figure 3) in response to nerve injury or any other noxious stimuli (26).

## Substance P Immunomodulation in Other Viral Infections and Latency

SP appears to contribute to other disease pathologies, such as respiratory syncytial virus and encephalo-myocarditis virus. It increases broncho-constriction and cardiac inflammation following infection (29, 30). SP is found to be directly related to inflammation. It can be used as a diagnostic and prognostic marker for COVID-19 infection. The role of SP in various pathologies is well-established, including in HIV-AIDS (31). Neuropeptide SP binds to NK-1R and accelerates HIV infection and inflammation in immune cells through CD163 receptor (32). Aprepitant, which is an NK-1R antagonist, blocks this inflammatory cascade and may serve as a therapeutic regimen against HIV infection (33). Involvement of SP has also been found in viral myocarditis, one of the main factors for cardiac arrest. The etiological factor for this infection is encephalao-myocarditis virus. NK-1R may also be targeted by specific antagonist drugs in this infection and they inactivate the SP signaling pathway and, hence, the inflammation (30, 34). SP has also been found to have a pathogenic role in rats infected with rat corona virus, parainfluenza virus 1 (35).

**Figure 4:**
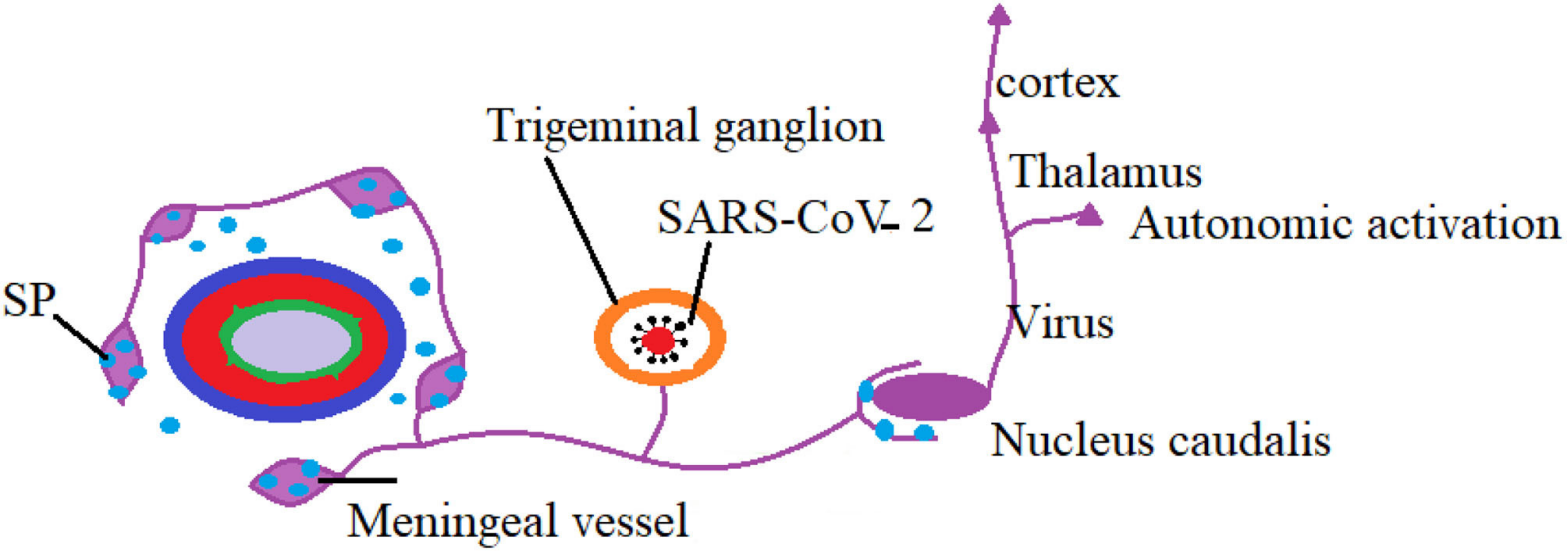
SARS-CoV-2 virus in trigeminal ganglion.

Herpes simplex virus type 1 (HSV-1) infects the ocular mucosal membrane and orofacial region and continues to infect the sensory neurons leading to a latent infection in TG (Figure 4). The virus reaches TG after passing through the axons and maintains a latent infection throughout the life of the infected person where it encodes latency associated transcript (LAT) (36). Reactivation of the virus was found more in TG as compared to brainstem in another study (37). We may imagine a similar mechanism and pathology in COVID-19 infection as well. Previous studies have shown that latent HSV-1 infection of TG can alter the expression of many neuronal genes, including those involved in the immune response, axonal remodeling, signal transduction, and gene expression (37). However, there is little evidence that HSV-1 LAT can affect the expression of neuropeptides in TG. The specificity of LAT action on TG may help in understanding the reason why the primary location for latent HSV1 infection is the TG (38).

Latent HSV-1 DNA has been detected in the CNS of human postmortem, and infection with HSV has been correlated with the development of neurodegenerative diseases. However, whether HSV can directly reactivate in the CNS and/or infectious virus can be transported to the CNS following reactivation in peripheral ganglia has been unclear. Viral proteins were detected in neurons of the TG. These results suggest that infectious virus is transported from the TG to the CNS following reactivation but does not exclude the potential for direct reactivation in the CNS (38).

SP is the first to react in response to a noxious stimulus. It is a rapid and immediate defense in the survival system. In experimental studies, NK-1R deficient mice exhibited reduced pulmonary inflammation as compared to the controls (39). Immune response prevents the host cells by fighting against the pathogen but if it continues uncontrolled, it may be fatal. It is known as “cytokine storming.” Inflammatory mediators continue to be secreted by immune cells and can cause acute respiratory distress syndrome (ARDS) in COVID-19 infection. So, it is not actually the pathogen that is fatal, but the cytokine storming. If prevented or reversed, it may save the infected patients (40, 41).

SP/NK-1R may control the breathing activity in neonates, evident from the raised immunohistochemical SP expression in the brainstem tissues after postmortem studies in control infants as compared to Sudden Infant Death syndrome (SIDS) victims in my previous study at Centro Lino Rossi, University of Milan, Italy (6, 16). SP expression was higher in sudden fetal deaths (16) and sudden death in adults (42). SP and NK-1R regulates the breathing and cardiovascular control in the medulla as a consequence of hypoxia. In a study conducted on SIDS victims, a significant decrease in NK-1R binding within medullary nuclei in SIDS was observed as compared to controls. Alteration in SP secretions and modulation may disturb the autonomic functionalities leading to lack of arousal and cause SIDS (43). It explains a possible mechanism of causality in elderly patients due to COVID-19. As a neuromodulator, SP dilates the vessels, causes smooth muscle contraction in respiratory walls, increases the excitatory potential by neurons, and increases vascular permeability (44) and saliva production. Under pathological conditions, it may cause broncho-constriction (45). My other study highlighted the fact that SP encoding gene TAC-1 has unconventional networking properties: being a singleton, small protein interaction network, and the members of tachykinin family have conserved amino acid sequences, which make it vulnerable to being a causative agent for various diseases, including fatal ones (46).

Reactivation occurs in Herpes and HIV but is not common in other types of viruses. It has not been speculated to exist in SARS-CoV-2, at least in a shorter duration of time. But if it proves to be the case then it may cause infection again at a later stage in life. Upon contracting the virus in orofacial parts, the virus reaches the TG through TrN and may reside there, taking control of the release of its peptides, particularly SP. The vaccines may also not be of much help in eradication of the disease (47). Antibodies generated as an immune response to weakened viruses may only fight with the new active viruses. It will not kill the already present latent, inactive viruses inside the TG or other cells. These latent viruses may escape the immune system and become a continuous threat for disease.

Antibody therapies such as passive immunization and Intravenous immunoglobulin therapy may also not be useful in these circumstances. In that case, the promising strategy would be the use of antiviral drugs, corticosteroids, and SP/NK-1 blockers to cease the inflammatory responses and attack the latent viruses as well. Although this possibility has limited chances, it cannot be ruled out totally due to its entry route through trigeminal nerves and ability to target TG like other latent viruses such as Herpes Simplex Viruses. We have to keep an eye on this phenomenon as well. Whether the virus becomes latent upon reaching TG or not is one aspect that needs to be explored, but there are more possibilities that this virus reaches TG, modulates SP release, and initiates inflammatory mechanisms.

Another compelling argument is that these viruses are highly mutating and transforming, making them a difficult target for vaccines. Vaccines are successful for those viruses that mutate less, have strictly humans as host, and have only one stable antigenic type. Common cold viruses, including SARS-CoV-2, have at least 100 antigenic types and a high mutation rate.

Serotonin and CGRP also co-exist with SP and should be explored as well. The abnormal release of SP from CN V may be associated with the acute respiratory symptoms in SARS-CoV-2 infection. SP may trigger the immune cells to release the inflammatory mediators in CNS as well as in the respiratory system, causing cytokine storming, inflammation, lung injury, and broncho-constriction. In complicated cases it may lead to cardiac failure. TG stimulation by virus may also increase the conductance of sodium ions and decrease the conductance of potassium ions in the cells leading to hypernatremia, hypokalemia, and dehydration. This electrolyte and fluid balance will exert pressure on the kidneys and pumping of the heart, causing failure and death. Maintaining electrolyte and fluid balance is necessary to manage these patients. Potassium and magnesium should be added in their dosage. High sunlight exposure and stress may also reactivate the virus. But all these phenomenon need to be evaluated and clinical trials are required.

## Data Availability Statement

The original contributions presented in the study are included in the article/supplementary material, further inquiries can be directed to the corresponding author/s.

## Author Contributions

RM has conceived the idea and proposed the main theory. MK, AB, NA, HN, LM, TA, AR, and RJ have critically evaluated, contributed in the write up and finalization of the manuscript.

## Conflict of Interest

The authors declare that the research was conducted in the absence of any commercial or financial relationships that could be construed as a potential conflict of interest.

## Publisher's Note

All claims expressed in this article are solely those of the authors and do not necessarily represent those of their affiliated organizations, or those of the publisher, the editors and the reviewers. Any product that may be evaluated in this article, or claim that may be made by its manufacturer, is not guaranteed or endorsed by the publisher.
